# Supplementary material for: Improvement in the Management of Oral Anticoagulation in Patients with Atrial Fibrillation in Primary Health Care
Source: Int J Environ Res Public Health. 2022 May 31;19(11):6746. doi: 10.3390/ijerph19116746 (PMC9180454; doi:10.3390/ijerph19116746)
Supplement: Supplementary file 1 [file ijerph-19-06746-s001.zip › ijerph-1681117-supplementary.pdf]

Supplementary file S1

GUIA RÁPIDA PARA EL MANEJO DE LOS ACOD EN AP

INICIO

1º- Abrir episodio de alteración de coagulación al respecto y asegurarnos de que la indicación sea adecuada y de la ausencia de contraindicaciones.

INDICACIONES: FA NO VALVULAR

1. **De inicio:**
   - Hipersensibilidad conocida o contraindicación AVK.
   - Antecedentes de Hemorragia intracerebral.
   - Ictus isquémico con criterios clínicos y de neuroimagen de alto riesgo de HIC.
2. **Tras AVK:**
   - Episodios tromboembólicos con AVK peses a INR bien controlados.
   - ACO con AVK en los que no es posible mantener un control de INR dentro de rango pese a un buen cumplimiento terapéutico*.
   - Imposibilidad de acceso al control de INR convencional.

CONTRAINDICACIONES:

COMUNES A TODOS LOS ACO:

- Falta de colaboración del paciente (demencia, trastorno psiquiátrico grave, alcoholismo, etc.).
- Hemorragia aguda (en las 2 semanas previas), incluyendo la intracraneal.
- Pericarditis aguda, endocarditis.
- Cirugía reciente (o programada) del sistema nervioso central.
- Traumatismo craneoencefálico significativo reciente.
- Hipertensión grave o no controlada.
- Enfermedades hepáticas o renales graves (riesgo de sangrado).
- Alteración de la hemostasia (coagulación, fibrinolisis, plaquetaria) hereditaria o adquirida con riesgo clínicamente relevante de hemorragia: por ejemplo, trombocitopenia significativa (plaquetas < 50.000/mm3).
- Embarazo.

ESPECÍFICAS DE ACODS

- Fibrilación auricular valvular (prótesis valvular metálica y Estenosis mitral moderada-severa (usualmente origen reumático).
- valvulopatía mitral reumática, otras valvulopatías graves).
- Función renal (ClCr):
  - < 30 ml/min: dabigatrán
  - <15 ml/min: apixaban, rivaroxaban y edoxaban.
- Insuficiencia hepática.
- No recomendados cuando hay alto riesgo de sangrado gastrointestinal. Recordar la indicación de gastroprotección (con IBP).

2º- Valoración de la función renal del paciente.

Estimación del filtrado glomerular (eFG) mediante la fórmula **MDRD o CKD-EPI** (calculadoras de función renal) o **Aclaramiento de creatinina** (Cockroft-Gault).

3º- Valoración función hepática del paciente.

- Según parámetros analíticos se considera alterada si: Bilirrubina > x 2 y Transaminasas (GOT; GPT) y FA > x 3 valores máximos del rango de normalidad.

4º- Revisar posibles Interacciones farmacológicas:

Interacciones		Dabigatrán	Rivaroxabán	Apixabán
↑ actividad anticoagulante	Antimicóticos azólicos, inhibidores proteasa	No asociar	No asociar	No asociar
	Dronedarona	No asociar	No asociar	
	Macrólidos	Precaución	Precaución	
	Amiodarona	Precaución (insuficiencia renal)		
	Quinidina	Precaución (insuficiencia renal)		
	Verapamilo	Precaución (110 mg/12 h, administrar juntos)		
↓ actividad anticoagulante	Carbamazepina, fenitoína, fenobarbital, rifampicina	No asociar	No asociar	Precaución
	Ciclosporina, tacrolimús	No asociar		
AINE		Precaución si vida media larga		
AAS o clopidogrel		Aumenta riesgo de sangrado pero sin interacción farmacocinética		
Otros AC		No asociar	No asociar	No asociar

5º- Explorar: Buscar signos de sangrado y valoración de TA y frecuencia cardiaca central.

6º- Elección y Ajuste de dosis por situaciones específicas: Edad, peso, función renal o tratamientos concomitantes.

ACOD	DABIGATRAN	RIVAROXABAN	APIXABAN	EDOXABAN
**Dosis Habitual.**	150 mg/12h	20 mg/24h	5 mg/12h	60 mg/24h
**Dosis Ajustada.**	110 mg/12h<br>• Edad ≥ 80 años.<br>• ERC moderada (ACr 30-50 ml/min) y/o 75-79 años con riesgo hemorrágico alto (peso <50Kg,AAG o AINE, etc)<br>• Trt con Verapamil.	15 mg/24h<br>• ERC moderada (ACr 30-50 mil/min).<br>• ERC grave (ACr 15-30 mil/min).	2.5 mg/12h<br>• Pacientes con al menos dos de los siguientes criterios:<br>- Edad ≥ 80 años.<br>- Peso ≤ 60Kg.<br>- Creatinina sérica ≥ 1.5 mg/dl<br>• ERC grave (ACr 15-30 mil/min).	30 mg/24h<br>• Peso ≤ 60Kg.<br>• ERC moderada (ACr 30-50 mil/min).<br>• ERC grave (ACr 15-30 mil/min).<br>Uso concomitante de<br>• Inh. GpP (Dronedarona, Ketoconazol, ciclosporina, eritromicina).

- **Documentación asociada:**

  a - Información al paciente por escrito.

  b - Informe para el visado de Inspección.

SEGUIMIENTO

FRECUENCIA:

Revisión consulta de enfermería	Revisión programada consulta médica
• A los 14 días del inicio<br>• Cada mes, durante los 3 primeros meses<br>• Después, cada 2 meses (individualizar según adherencia, edad, comorbilidad, polimedicación, riesgo de sangrado, etc.)	• Al mes y a los 3 meses del inicio<br>• A los 6 meses (hemograma, función renal)<br>• Posteriormente cada 3-6 meses (individualizar)<br>• Siempre que se considere en la consulta de enfermería<br>• Continuar según protocolo de crónicos<br>• Las visitas deben coincidir en la agenda con las de enfermería de forma coordinada

CONTENIDOS

- Adherencia al tratamiento (test de Morisky-Green)
- Posibles efectos adversos (dispepsia, sangrado en cualquier localización, otros)
- Control de la tensión arterial y de la frecuencia cardiaca
- Control de hemograma, función renal y hepática (a los 6 y a los 12 meses) o si se presentan enfermedades intercurrentes
- Sintomatología en relación con la arritmia y/o enfermedad de base
- Educación/información paciente y familia
- Refuerzo positivo (optimizar adherencia)

CONTROLES ANALÍTICOS:

FUNCIÓN RENAL:

- **Mínimo al inicio y anual.**
- **En pacientes mayores (> 75 años) o con comorbilidad: Mínimo cada 6 m**
- **Según eFG:**
  - **IRC Moderada (GRF 30-60): 6 M**
  - **IRC Severa (GRF 15-30): 3 M**
- **Y, adicionalmente, en situaciones clínicas que permitan sospechar un deterioro de la función renal.**

HEMOGRAMA Y FUNCIÓN HEPÁTICA: Anualmente.

NO SE PRECISA MEDIR EL NIVEL DE ANTICOAGULACIÓN.

Supplementary file S2

GUIA RÁPIDA PARA EL MANEJO DE LOS AVK EN AP

INICIO

1º- Abrir episodio de alteración de coagulación al respecto (B86) y asegurarnos de que la indicación sea adecuada y de la ausencia de contraindicaciones.

INDICACIONES: FA con criterio de ACO tras evaluación individual según Riesgo tromboembólico y hemorrágico por Escalas CHADs-VASc y HAS-BLED (Disponibles en protocolos de OMI).

- **Criterio de Indicación utilizado en OMI: Guías Europeas 2010:**

- **Score 0:** Riesgo bajo → Nada o AAS (preferiblemente nada)
- **Score 1:** Riesgo moderado → Anticoagulación o AAS (preferiblemente **anticoagulación**)
- **Score ≥ 2:** Riesgo alto → **Anticoagulación**

  - **La duración será indefinida y el rango habitual de INR (2-3).**

CONTRAINDICACIONES:

COMUNES A TODOS LOS ACO:

- Falta de colaboración del paciente (demencia, trastorno psiquiátrico grave, alcoholismo, etc.).
- Hemorragia aguda (en las 2 semanas previas), incluyendo la intracraneal.
- Pericarditis aguda, endocarditis.
- Cirugía reciente (o programada) del sistema nervioso central.
- Traumatismo craneoencefálico significativo reciente.
- Hipertensión grave o no controlada.
- Enfermedades hepáticas o renales graves (riesgo de sangrado).
- Alteración de la hemostasia (coagulación, fibrinolisis, plaquetaria) hereditaria o adquirida con riesgo clínicamente relevante de hemorragia: por ejemplo, trombocitopenia significativa (plaquetas < 50.000/mm3).
- Embarazo (durante el 1º trimestre y desde la semana 36).

2º- Revisar posibles Interacciones farmacológicas. (Anexos)

3º- Cargar la prescripción del anticoagulante en el episodio creado.

4º- Informar al paciente de forma oral y por escrito de:

- Recomendaciones Generales.
- Recomendaciones alimenticias.
- Medidas en caso de hemorragia.

Disponibles en planes personales, Tratamiento anticoagulante, actividades realizadas por todos o enfermería.

5º- Derivación a hematología para Inicio de la ACO.

a) **En días laborables:** Se remitirá hoja de consulta y petición de hemostasia básica con tubo de coagulación (tapón azul) y tubo de hemograma (tapón malva), junto con el resto de hojas de ACO de pacientes cuyo control del TAO se realice en el servicio de Hematología del HUMS.
b) **En días no laborables** (sábados y días festivos) se valorará si la necesidad de ACO puede esperar al día laborable más próximo o se remitirá al paciente al servicio de Urgencias del HUMS para iniciar ACO.

En la hoja de consulta se hará constar al menos la indicación de ACO, peso del paciente, comorbilidades, riesgo trombótico y hemorrágico y medicación del paciente que pueda interferir con el fármaco AVK.

6º - Recaptación del paciente estabilizado:

Se procederá a la misma tras notificación por hematología de la consecución de estabilización del INR (al menos 3 en rango) mediante indicación de **"Transferencia de control de TAO a AP"** en los comentarios de la dosificación por sistema Gota enviados por FAX al Centro de Salud.

El equipo de urgencias (DUE) que reciba dichos informes por la tarde, se encargará de la supervisión de la citación de los pacientes transferidos en la agenda de los médicos responsables y de la comunicación a los pacientes cuando recojan la dosificación.

Cada equipo MAP/DUE procederá a la inclusión del paciente en TAOnet según "Manual de Uso de la integración de TAONET con OMI" pag. 21 y a la organización de su posterior seguimiento.

7º- Cumplimentación del ``Consentimiento informado´´ e inclusión del paciente en cartera de servicios. En protocolos de OMI.

SEGUIMIENTO

Las consultas de seguimiento deben ser conjuntas (medicina y enfermería).

FRECUENCIA:

Según resultados de INR.

CONTENIDOS:

- **Valoración de Adherencia** (Test de Morisky-Green).
- **Educación del paciente:** Interacciones alimenticias y farmacológicas, Efectos secundarios del tratamiento con ACO, Refuerzo positivo para optimización de adherencia.
- **Sintomatología** en relación con arritmia y complicaciones (Trombóticas o hemorrágicas).
- **Exploración:** Control de TA y frecuencia cardiaca o complicaciones.
- **Evaluación INR:** Técnica, dosificación y próxima citación.

ACTITUD ANTE UN INR FUERA DE RANGO:

1- Interrogar: Buscar la causa y si ésta se va a mantener.

- Causas de INR fuera de rango:

INR < 1.5	INR > 5
Olvidos o errores.	Errores de dosificación.
Problemas en la extracción de la muestra.	Enfermedades intercurrentes (catarros..).
Cambios en la dieta (vegetales).	Cambios en la dieta (Alcohol).
Aumento de la actividad física.	Disminución en la actividad física.
Inicio de medicación inhibidora.	Inicio de medicación potenciadora.
Retirada de medicación potenciadora.	Hepatopatía.
Aumento de peso.	Pérdida de peso.
Mejoría del estado general.	Descompensación cardiaca.
Técnica de punción.	Técnica de punción.

2- Ajustar la dosis:

Es potestad exclusiva del facultativo. Se podrá realizar por TAONET o manualmente. Se utilizará la DTS (Dosis Total Semanal) y el intervalo de seguimiento dependerá de las cifras obtenidas.

- **Criterio de ajuste de dosis según resultado del INR:**

RANGO 2-3	ACTITUD	RANGO 2.5-3.5
**1.1-1.49**	↑ DTS 10-20% si es posible 1º día<br>Control en una semana.<br>Si riesgo elevado de trombosis + HBPM hasta INR en rango.	**1.1-1.99**
**1.5-1.9**	↑ DTS 5-10%<br>Control en 2 semanas.	2.00-3.9
**2.0-3.0**	Mantener DTS<br>Control en 4-6 semanas.	**2.5- 3.5**
3.1-3.99	↓ DTS 10%<br>Control en 2 semanas.	3.5-4.5
**4.00-5**	↓DTS 15-20%<br>Control en 2 semanas.	4.5-5.0
5.0-7.0	No tomar dosis ese día.<br>↓DTS 20-25 %<br>Control en 1 semana. **POSIBILIDAD DE REMITIR A HEMATOLOGIA**<br>Si riesgo de hemorragia: 2-5 mg Vit K (0.2-0.5 cc amp. Konakion®)	5.0-7.0
> 7	No tomar dosis ese día + 2-5 mg Vit K (0.2-0.5 cc amp. Konakion®)<br>↓DTS 20-25 %<br>Control en 1 semana<br>**POSIBILIDAD DE REMITIR A URGENCIAS HOSPITAL**	> 7

Ante toda hemorragia en la que no se consiga realizar hemostasia se derivará a urgencias independientemente del INR.

CAUSAS DE DERIVACIÓN A HEMATOLOGIA:

1- Razón Normalizada Internacional (INR) ≥5 si se considerara oportuno (hasta estabilización de INR).
2- Cambios clínicos relacionados con cualquiera de las contraindicaciones al uso de acenocumarol o warfarina.
3- Hospitalización.
4- Fin del tratamiento.
5- Embarazo.
6- Cáncer. Procesos neoplásicos en tratamientos quimioterápicos.
7- Ulceras gastrointestinales.
8- Intervenciones quirúrgicas (pendiente de protocolización).
9- Hipertensión grave.
10- Eventos tromboembólicos de repetición con INR en rango. Se remitirá nota por parte del servicio de Hematología a AP para valorar indicación de ACOD.
11- Inicio de tratamiento con dosis altas de AINES, miconazol, fenilbutazona, ácido acetil salicílico, salicilatos.
12- Fallecimiento.

SITUACIONES DE PLANTEAMIENTO DE ACOD TRAS AVK:

- Episodios tromboembólicos con AVK peses a INR bien controlados.
- ACO con AVK en los que no es posible mantener un control de INR dentro de rango (TRT directo < 60% o por Rosendaal > 65%) pese a un buen cumplimiento terapéutico*.
- Imposibilidad de acceso al control de INR convencional.

TRATAMIENTOS QUE NO INTERFIEREN DE FORMA IMPORTANTE CON AVK

**ANALGÉSICOS/AINES**	Paracetamol,<br>Dextropropoxifeno,<br>Codeína,<br>Tramadol	Diclofenaco,<br>Ibuprofeno,<br>Fenoprofen,<br>Nabumetona,<br>Celecoxib,<br>Rofecoxif<br>Glucocorticoide
**ANTIMICROBIANOS/ANTIPARASITARIOS**	Amoxicilina, Ampicilina<br>Amoxi/clavulanico<br>Josamicina, Cloxamicina<br>Ac pipemidico<br>Norfloxacino, Ofloxacino, Levofloxacino<br>Mebendazol	
**HIPOLIPEMIANTES**	Pravastatina, Pitavastatina?	
**HIPOGLUCEMIANTES**	Insulina<br>ADO menos sulfunilureas	
**ANTIGOTOSOS**	Alopurinol<br>Colchicina	
**AP CARDIOVASCULAR**	Nifedipino<br>Verapamil, Diltiazem<br>Nitritos<br>Atenolol, Bisoprolol, Metoprolol<br>Metildopa, Prazosina<br>Captopril, Enalapril<br>Furosemida, HCTZ, Indapamida<br>Digoxina	
**AP DIGESTIVO**	ANTIACIDOS: Almagato, Magaldrato<br>Pentoprazol, Omeprazol?	
	LAXANTES: Lactulosa, plantago, glicerina, agar	
	ANTIEMÉTICOS: Metoclopramida, Clebopride, Domperidona.<br>Loperamida	
**AP RESPIRATORIO**	ANTITUSIVOS: Codeina	
	MUCOLÍTICOS: Sin asociaciones	
	BRONCODILATADORES: Todos	
**SISTEMA NERVIOSO**	BZD: Diazepam, Clorazepato	
	ANTIDEPRESIVOS: Imipramina, Citalopram	
	ANTIPARKINSONIANOS: Mianserina, Biperideno, Levodopa	
	ANTIMIGRAÑOSOS: Ergotamina.	
**ANTIHISTAMÍNICOS**	Todos	

TRATAMIENTOS QUE INTERFIEREN CON AVK

POTENCIADORES		INHIBIDORES	
**FUERTES**	**DEBILES**	**FUERTES**	**DEBILES**
**ANALGÉSICOS-AINEs**			
-AAS	-SULFINPIRAZONA<br>-INDOMETACINA<br>-NAPROXENO<br>-DIPIRONAS (metamizol)<br>-PIROXICAM		
**ANTIMICROBIANOS Y ANTIPARASITARIOS**			
- COTRIMOXAZOL (TMT-SMX)<br>-TETRACICLINAS<br>-IMIDAZOLES (Metronidazol, Ketoconazol, ornidazol)	-QUINOLONAS<br>-MACRÓLIDOS<br>-ISONIAZIDA<br>-CLINDAMICINA	-RIFAMPICINA	
**HIPOLIPEMIANTES**			
-FIBRATOS	-SIMVASTATINA	-COLESTIRAMINA<br>-COLESTIPOL	
**PSICOFÁRMACOS**			
	-ANTIDEPRESIVOS TRICÍCLICOS<br>-ISRR (Paroxetina,fluoxetina)		
**OTROS**			
-AMIODARONA	-CLORPROMACINA<br>-ANTIULCEROSOS (cimetidina, ranitidina,omeprazol,lansoprazol)<br>-TIROXINA<br>-TAMOXIFENO<br>-DISULFIRAN	-BARBITÚRICOS<br>-FENITOINA<br>-POLESTRIENO<br>-CARBAMAZEPINA<br>-SUPLEMENTOS VITAMINA K	-AMINOGLUTETIMIDA<br>-CICLOSPORINA A<br>-TICLOPIDINA<br>-RITONAVIR

Supplementary file S3

GOBIERNO DE ARAGON

salud servicio aragonés de salud

PROYECTO DE MEJORA DE LA CALIDAD 437

1. TÍTULO

OPTIMIZACION DE LA ANTICOAGULACION ORAL DE LA FIBRILACION AURICULAR EN ATENCION PRIMARIA

2. RESPONSABLE .... AFRICA GARCIA ROY
   - Profesión .... MEDICO/A
   - Centro .... CS LAS FUENTES NORTE
   - Localidad .... ZARAGOZA
   - Servicio/Unidad .. ATENCION PRIMARIA
   - Sector .... ZARAGOZA 2

3. OTROS COMPONENTES DEL EQUIPO DE MEJORA.
   - SARSA GOMEZ ANA. MEDICO/A. CS LAS FUENTES NORTE. ATENCION PRIMARIA
   - BELLOSO RODANES MARIA ISABEL. MEDICO/A. CS LAS FUENTES NORTE. ATENCION PRIMARIA
   - SANCHEZ MARTINEZ ANA ISABEL. MEDICO/A. CS LAS FUENTES NORTE. ATENCION PRIMARIA
   - URIEL PEREZ JOSE ANTONIO. MEDICO/A. CS LAS FUENTES NORTE. ATENCION PRIMARIA
   - EZQUERRO CORDON ASCENSION. ENFERMERO/A. CS LAS FUENTES NORTE. ATENCION PRIMARIA
   - LAMANA LASHERAS MARIA JOSE. ENFERMERO/A. CS LAS FUENTES NORTE. ATENCION PRIMARIA
   - VALLES LOBATO MARIA JOSE. ENFERMERO/A. CS LAS FUENTES NORTE. ATENCION PRIMARIA

4. PROBLEMA U OPORTUNIDAD DE MEJORA SELECCIONADA. La Fibrilación auricular (FA) es un importante problema de Salud Pública por prevalencia y morbimortalidad asociada, fundamentalmente por eventos tromboembólicos, donde la anticoagulación oral (ACO) es la piedra angular del tratamiento, al ser la única capaz de reducirlos y que en la actualidad ofrece diferentes alternativas, Dicumarínicos y Anticoagulantes orales directos (ACOD).
Por otra parte, los pacientes con FA son controlados mayoritariamente en Atención Primaria (AP) y son frágiles por edad avanzada, comorbilidad asociada y polifarmacia. Por tanto somos responsables de garantizar la opción más favorable de tratamiento efectivo en un problema de gran magnitud y en una población especialmente vulnerable.
En el elevado número de pacientes con FA atendidos en nuestro centro de salud (CS) se objetiva un infratratamiento de ACO, controles subóptimos de los tratamientos con acenocumarol y alto porcentaje de controles desde hematología.
Esto supone un empeoramiento de nuestra calidad asistencial por la pérdida del efecto protector antitrombótico perseguido y de la continuidad de seguimiento.

5. RESULTADOS QUE SE ESPERA CONSEGUIR. Vistos los resultados de nuestro estudio de línea de mejora del 2015 sobre ``Características clínico- epidemiológicas y de manejo del paciente con FA atendido en el CS Las Fuente Norte`` nos planteamos como objetivo general optimizar el manejo de su anticoagulación oral, equiparándolos al menos con los observados a nivel nacional, mediante los siguientes objetivos específicos:
I. Asegurar la evaluación del riesgo tromboembólico y hemorrágico a todo paciente con FA seguido en nuestro CS.
II. Garantizar ACO a todo paciente con FA que tenga indicación para la misma.
III. Garantizar la calidad de la ACO ofreciendo la opción más adecuada de las disponibles según la legislación vigente (Informe de Posicionamiento terapéutico del Ministerio).
IV. Asumir el mayor control posible de la ACO desde AP.

6. MÉTODO Y ACTIVIDADES PARA MEJORAR. Tras la divulgación al equipo de los resultados del estudio del 2015, se determinarán las debilidades y los objetivos a conseguir, por comparativa cuando se disponga de un indicador de referencia nacional o por consenso del equipo en el caso de que este dato no esté disponible.
Se facilitarán los listados de pacientes con FA por CIAS y se procederá a la capacitación del equipo mediante talleres impartidos por los componentes de la línea de mejora, según cronograma preestablecido, con soporte por escrito de material de consulta rápida.
Se solicitarán a los servicios responsables las adaptaciones pertinentes del soporte informático para la puesta en marcha del Protocolo de manejo de ACOD del CS Las Fuentes Norte (diversificación de episodios de anticoagulación B-83, información por escrito para el paciente, adaptación de los sistemas de seguimiento para control de adherencia terapéutica por Morisky-Green, etc) así como cauces de comunicación fluida con las especialidades implicadas.
La reevaluación se realizará repitiendo la metódica del estudio previo observacional/retrospectivo, con datos de base administrativa y de auditoria de historia clínica, ampliado en las variables añadidas, de la población con registro de episodio de FA y seguimiento continuado, de los 22513 usuarios del CS.
Las fuentes de información serán la historia clínica para la variable principal y comorbilidades, receta electrónica para los tratamientos asociados y los programas informáticos TAOnet y Gota para la evaluación de los INR en AP y hematología respectivamente.
Para el análisis de resultados se empleará el paquete estadístico SPSS.
Se suplementará con evaluación mediante encuesta estructurada a los pacientes en tratamiento con ACOD.
Las conclusiones servirán para la definición de nuevas líneas de mejora.

7. INDICADORES, EVALUACIÓN Y SEGUIMIENTO. Abordando los objetivos específicos mencionados:
I- I1. Porcentaje de pacientes con registro en historia clínica de Riesgo tromboembólico y hemorrágico por escalas específicas al respecto (CHADs-VASc Y HAS-BLED).
I2. Porcentaje de pacientes con indicación de ACO por CHADs-VASc = 2 que la siguen.
I3. Porcentaje de pacientes con Antiagregación oral como prevención primaria.
I4. Porcentaje de pacientes en tratamiento con acenocumarol controlados en AP con control óptimo por Tiempo en rango terapéutico directo (TRT) = 60% en los últimos 6 meses.
I5. Porcentaje de pacientes seguidos en hematología con últimos 3 INR en rango.
I6. Porcentaje de pacientes en tratamiento con acenocumarol seguidos por AP.
I7. Porcentaje de pacientes en tratamiento con ACOD incluidos en protocolo específico de manejo.

1. TÍTULO

OPTIMIZACION DE LA ANTICOAGULACION ORAL DE LA FIBRILACION AURICULAR EN ATENCION PRIMARIA

I8. Grado de conocimiento, adherencia y satisfacción de los pacientes en tratamiento con ACOD.
Dichos indicadores se integraran en las actividades previstas según diagrama de flujo previamente establecido.

8. DURACIÓN Y CALENDARIO PREVISTOS. De octubre 2016 a junio de 2017.

9. OBSERVACIONES. Dado el ambicioso contenido del proyecto y en base a la limitación de los recursos humanos disponibles, el proyecto debiera desarrollarse secuencialmente durante más de un año, con las ampliaciones pertinentes según resultados y objetivos propuestos.

10. ENFOQUE PRINCIPAL. Guía, protocolo, vía clínica o procedimiento basado en la evidencia

POBLACIÓN DIANA
- ·EDAD. Adultos
- ·SEXO. Ambos sexos
- ·TIPO DE POBLACIÓN O PACIENTES. Enfermedades del sistema circulatorio

INFORMACIÓN COMPLETADA SOBRE EL PROYECTO

VALORACIÓN DEL PROYECTO EN RELACIÓN A:
1. Mejorar resultados de la atención. .... 4 Mucho
2. Dirminuir riesgos derivados de la atención. .... 4 Mucho
3. Mejorar utilización de recursos. .... 4 Mucho
4. Garantizar derechos de los usuarios (y > satisfacción). ... 4 Mucho
5. Atender expectativas de los profesionales. .... 4 Mucho
